# Remarks on the Uses of Papine

**Published:** 1886-10

**Authors:** 


### *REMARKS ON THE USES OF PAPINE.*

Dr. Wm. J. Crittenden, in a communication to the *Virginia Medical Monthly* says: During January, 1886, I was called to see a lady suffering with acute peritonitis. She assured me that she could not use opium, as she had tired of it previously. But I gave her one-eighth grain of morphia sulphate and one 1-120th grain of atropia sulphate, hypodermically, and in a few minutes the depressing effects were noted, both upon the respiration and circulation; the pupils also became visibly contraced. I then tried the various usual substitutes for morphia in succession, but to no effect. I determined to try papine; but not being able to give it by the mouth on account of nausea, and as she objected to the use of the hypodermic needle, I gave her two-drachms per rectum, and repeated it in one hour. The result was that she sank into a quiet peaceful sleep, which lasted for several hours. During the remainder of her sickness I gave her papine, with the most gratifying results. As soon as her stomach would retain it, I gave it to her by the mouth in one-drachm doses.

I have also used papine in a case of uterine cancer, in lieu of morphia. In cases which patients have been taking morphia until it has lost it anodyne influence, papine is well adapted.

In pneumonitis, pleuritis and bronchitis I have found papine to answer an excellent purpose. In dysentery it is useful, both as an anodyne and in relieving the tenesmus. In the diarrhœa of children I frequently combine with it bismuth subnitrate and prepared chalk. I have used it also in cystitis. In neuralgia, when I wish an anodyne, I use papine. As an anodyne it is equal, if not superior, to morphia; and I have never yet seen any unpleasant effects from it use. As a hypnotic I find it to be an agent of great value.

It is inferior to bromida when we simply wish the effect of a hypnotic. But it fulfills the indications when we wish a decided anodyne as well as a hypnotic influence.

I trust that the readers of the *Virginia Medical Monthly* may give this drug a trial, as I feel they will be amply repaid for their trouble.

